# Genome stability of the vaccine strain VACΔ6

**DOI:** 10.18699/VJGB-22-48

**Published:** 2022-07

**Authors:** R.A. Maksyutov, S.N. Yakubitskiy, I.V. Kolosova, T.V. Tregubchak, A.N. Shvalov, E.V. Gavrilova, S.N. Shchelkunov

**Affiliations:** State Research Center of Virology and Biotechnology “Vector”, Rospotrebnadzor, Koltsovo, Novosibirsk region, Russia; State Research Center of Virology and Biotechnology “Vector”, Rospotrebnadzor, Koltsovo, Novosibirsk region, Russia; State Research Center of Virology and Biotechnology “Vector”, Rospotrebnadzor, Koltsovo, Novosibirsk region, Russia; State Research Center of Virology and Biotechnology “Vector”, Rospotrebnadzor, Koltsovo, Novosibirsk region, Russia; State Research Center of Virology and Biotechnology “Vector”, Rospotrebnadzor, Koltsovo, Novosibirsk region, Russia; State Research Center of Virology and Biotechnology “Vector”, Rospotrebnadzor, Koltsovo, Novosibirsk region, Russia; State Research Center of Virology and Biotechnology “Vector”, Rospotrebnadzor, Koltsovo, Novosibirsk region, Russia

**Keywords:** vaccinia virus, transient dominant selection, targeted gene inactivation, genome stability, вирус осповакцины, временная доминантная селекция, направленная инактивация генов, стабильность генома

## Abstract

Due to cessation of mass smallpox vaccination in 1980, the collective immunity of humans against orthopoxvirus infections has virtually been lost. Therefore, the risk of spreading zoonotic human orthopoxvirus infections caused by monkeypox and cowpox viruses has increased in the world. First-generation smallpox vaccines based on Vaccinia virus (VAC) are reactogenic and therefore not suitable for mass vaccination under current conditions. This necessitates the development of modern safe live vaccines based on VAC using genetic engineering. We created the VACΔ6 strain by transient dominant selection. In the VACΔ6 genome, f ive virulence genes were intentionally deleted, and one gene was inactivated by inserting a synthetic DNA fragment. The virus was passaged 71 times in CV-1 cells to obtain the VACΔ6 strain from the VAC LIVP clonal variant. Such a long passage history might have led to additional off-target mutations in VACΔ6 compared to the original LIVP variant. To prevent this, we performed a genome-wide sequencing of VAC LIVP, VACΔ6, and f ive intermediate viral strains to assess possible off-target mutations. A comparative analysis of complete viral genomes showed that, in addition to target mutations, only two nucleotide substitutions occurred spontaneously when obtaining VACΔ4 from the VACΔ3 strain; the mutations persisting in the VACΔ5 and VACΔ6 genomes. Both nucleotide substitutions are located in intergenic regions (positions 1431 and 189738 relative to LIVP), which indicates an extremely rare occurrence of off-target mutations when using transient dominant selection to obtain recombinant VAC variants with multiple insertions/deletions. To assess the genome stability of the resulting attenuated vaccine strain, 15 consecutive cycles of cultivation of the industrial VACΔ6 strain were performed in 4647 cells certif ied for vaccine production in accordance with the “Guidelines for Clinical Trials of Medicinal Products”. PCR and sequencing analysis of six DNA fragments corresponding to the regions of disrupted genes in VACΔ6 showed that all viral DNA sequences remained unchanged after 15 passages in 4647 cells.

## Abstract (ru)

В связи с прекращением после 1980 г. массовой противооспенной вакцинации в настоящее время практически полностью утрачен коллективный иммунитет человеческой популяции к ортопоксвирусным инфекциям. Вследствие этого увеличилась опасность распространения в мире зоонозных ортопоксвирусных инфекций человека, обусловленных вирусами оспы обезьян или оспы коров. Противооспенные вакцины первого поколения на основе вируса осповакцины (Vaccinia virus, VAC) являются реактогенными и поэтому в современных условиях не пригодны для массовой вакцинации. Это обусловливает необходимость разработки современных безопасных живых вакцин на основе VAC с применением методов генетической инженерии. С использованием метода временной доминантной селекции нами создан штамм VACΔ6, в геноме которого пять генов вирулентности направленно делетированы, а один ген инактивирован встройкой синтетического фрагмента ДНК. В процессе получения штамма VACΔ6 из клонового варианта VAC LIVP вирус прошел 71 пассаж в культуре клеток CV-1. Такая длинная пассажная история могла привести к дополнительным нецелевым изменениям в геноме штамма VACΔ6 относительно исходного LIVP. Поэтому для оценки возможных нецелевых изменений провели полногеномное секвенирование VAC LIVP, VACΔ6 и пяти промежуточных штаммов вируса. Сравнительный анализ полных вирусных геномов показал, что, помимо целевых нарушений, спонтанно произошли только две нуклеотидные замены при получении VACΔ4 из штамма VACΔ3 и сохранившиеся в геноме VACΔ5 и VACΔ6. При этом обе эти замены находятся в межгенных участках (позиции 1431 и 189738 относительно штамма LIVP), что указывает на крайне редкое возникновение нецелевых изменений при использовании методики временной доминантной селекции для получения рекомбинантных VAC со множественными встройками/делециями. Для выяснения стабильности генома полученного аттенуированного вакцинного штамма и в соответствии с «Руководством по проведению клинических исследований лекарственных средств…» выполнено 15 последовательных циклов культивирования производственного штамма вируса VACΔ6 в культуре клеток 4647, аттестованной для производства вакцины. ПЦР-анализ и секвенирование шести фрагментов ДНК, соответствующих районам нарушаемых генов VACΔ6, показали, что после 15 пассажей в культуре клеток 4647 все последовательности вирусной ДНК остались неизменными.

## Introduction

In 1980, after the declaration of smallpox eradication by the World Health Organization (WHO), it was recommended to stop vaccinating people against this extremely dangerous disease. This decision was due to the fact that Vaccinia virus (VAC) vaccinations can cause severe post-vaccination complications and even death in some cases (Smallpox and its Eradication, 1988; Kretzschmar et al., 2006).

Due to cessation of vaccination against smallpox, there are fewer people with specific immunity against the disease every year. This makes humans susceptible not only to possible variola virus infection but also other closely related orthopoxviruses, whose natural reservoir is various animals, primarily rodents. These viruses include monkeypox and cowpox, which cause smallpox-like diseases in animals and humans. The spread of these viruses in the human population can potentially lead to their adaptation to the host antiviral defense and occurrence of viral variants that are epidemically dangerous for humans (Shchelkunov, 2013). In recent years, unusually large outbreaks of orthopoxvirus infections have been recorded among humans in various regions of the world (Singh et al., 2012; Nolen et al., 2016; Reynolds et al., 2019).

Vaccination is the only effective method to combat the growing threat of human orthopoxvirus infections (Moss, 2011; Shchelkunov, 2011). The accumulation of immunodeficiency states in the human population in recent decades has led to contraindication of the use of classical live VAC-based vaccines for mass vaccination, since it can cause a large number of adverse reactions and more severe manifestations than those observed during the smallpox eradication campaign (Albar- naz et al., 2018; Shchelkunov, Shchelkunova, 2020). Therefore, there is an urgent need to develop modern orthopoxvirus vaccines that should be both much safer than previous generations of smallpox vaccines and highly immunogenic, thus providing reliable protection against viral infection.

The first attenuated VAC strains were obtained by serial passage of the virus in heterologous host cells: the MVA strain was generated upon 572 passages of VAC Ankara in primary chicken embryo fibroblasts (Volz, Sutter, 2017), the LC16m8 strain was produced after 45 passages of VAC Lister in primary rabbit kidney cells (Kidokoro, Shida, 2014; Eto et al., 2015).

VAC MVA attenuation was due to spontaneous extended deletions and mutations in the viral genome that affect not only virulence genes but also genes responsible for viral replication and the range of virus-sensitive hosts (Blanchard et al., 1998; Drexler et al., 1998). MVA lost its ability to form infectious progeny in most mammalian cell cultures, including human cells. Many MVA genes are expressed in these cell cultures; however, only immature virions are produced. MVA has retained its immunogenic properties as a smallpox vaccine; however, in order to achieve a sufficient immune response, the virus should be administered at higher doses and multiple times compared to the classical vaccine (Sanchez-Sampedro et al., 2015).

Attenuation of the VAC LC16m8 clonal variant is due to a single nucleotide deletion in the B5R gene encoding the envelope protein of extracellular virions. The mutation creates a translational frameshift. In mammalian cells, LC16m8 produces infectious viral particles with a reduced ability to spread in both cell cultures and infected/vaccinated organisms. LC16m8 is less attenuated than MVA and it is a replicationcompetent vaccine (Sanchez-Sampedro et al., 2015; Albarnaz et al., 2018).

Advances in genetic engineering techniques have made it possible to create modified VAC variants by either introducing the desired nucleotide sequences into the viral genome or deleting/ disrupting viral genes. One of the most promising areas is the use of genetic engineering to develop highly attenuated VAC variants with the same levels of immunogenicity and protectiveness but lower pathogenicity compared to those of the classical smallpox vaccine (Shchelkunov, Shchelkunova, 2020).

Whole-genome sequencing of various strains and different orthopoxvirus species that are pathogenic to humans, accumulation of data on functions of numerous viral genes, and development of methods for introducing targeted changes into the viral genome have made it possible to formulate and implement a new approach to the development of highly attenuated VAC variants. The approach involves strictly localized sequential deletion/inactivation of individual virulence genes without affecting virus replication in a cell culture and the range of virus-susceptible hosts (Yakubitskiy et al., 2015).

We have previously obtained live attenuated vaccine strain VACΔ6 against smallpox and other human orthopoxvirus infections by targeted sequential inactivation of individual viral genes (Yakubitskiy et al., 2015, 2016). We used transient dominant selection to produce this strain (Falkner, Moss, 1990). Each stage of the method involved multiple cycles of viral reproduction (passages) in a cell culture. By that time, no information on how these procedures can affect preservation of the nucleotide sequence of the large VAC DNA genome had been available.

In this regard, the aim of the work was to study the degree of VAC genome preservation during generation of the VACΔ6 strain from the parental LIVP strain by whole-genome sequencing. Another equally important issue was the genomic stability of the industrial vaccine strain VACΔ6 after 15 passages in 4647 cells used specifically for production of the smallpox vaccine

## Materials and methods

Viruses and cell cultures. We used clone 14 of the VAC LIVP (LIVP) strain, which had been previously obtained by limiting dilution with threefold plaque purification using an agarose overlay (Yakubitskiy et al., 2015), and mutant LIVP-derived VAC variants with inactivation of the target genes (Yakubitskiy et al., 2015, 2016). Viruses were grown and titrated in African green monkey kidney cell lines CV-1 and 4647 from the cell culture collection of the State Research Center of Virology and Biotechnology “Vector” of Rospotrebnadzor. Cell line 4647 was certified by L.A. Tarasevich State Institute of Standardization and Control of Biomedical Preparations in accordance with the requirements of Guidance document 42-28-10-89 and recommended for production of preventive medical immunobiological preparations (MIBPs) (protocol No. 14 of the meeting of the Academic Council of L.A. Tarasevich State Institute of Standardization and Control of Biomedical Preparations dated October 28, 2003; protocol No. 9 of the MIBP Committee dated November 20, 2003).

Oligonucleotide primers. Oligonucleotides for PCR analysis of inactivated viral gene regions (Table 1) were synthesized at the Institute of Chemical Biology and Fundamental Medicine of the Siberian Branch of the Russian Academy of Sciences. The oligonucleotide primers were designed using Oligo software version 3.3 (Borland International, USA).

Virus cloning. Prior to cloning, viral suspension was sonicated, and the titer was estimated by plaque assay in CV-1 cells. The virus was cloned in 6-well culture plates by agarose overlay plaque assay. For this, monolayer CV-1 cells were infected with 10–20 PFU/well of a viral suspension. The virus was adsorbed for 60 min at 37 °C and 5 % CO2. The medium with unabsorbed virus was replaced with 2 ml/well of DMEM medium (BioloT, Russia) supplemented with 2 % fetal bovine serum (FBS) (HyClone, USA) containing 1 % low melting agarose. After agarose solidified, the plates were incubated in a thermostat for 48 h at 37 °C and 5 % CO2. Then, 1.5 ml/ well of a 0.05 % neutral red solution in DMEM medium was added, and plates were incubated for 1 h at 37 °C and 5 % CO2. The neutral red solution was then removed, and individual plaques were collected using an automatic pipette and then transferred to 100 μl of DMEM medium supplemented with 2 % FBS. The resulting virus sample after a single freeze–thaw cycle was inoculated onto CV-1 cell monolayers in individual wells of 6-well plates containing 1 ml/well of DMEM medium with 2 % FBS. Cells were incubated for 48–72 h at 37 °C and 5 % CO2. The resulting virus suspension was frozen–thawed twice and used to further produce the clone at a working titer of 106–107 PFU/ml.

Generation of viral variants with targeted gene deletions. Recombinant VAC variants were obtained in CV-1 cells by cationic lipid-mediated transfection with Lipofectin Reagent (Invitrogen, USA) and a selective medium containing mycophenolic acid (MPA), xanthine, and hypoxanthine (Sigma, USA). For this, CV-1 cells at 80–90 % confluence in 6-well culture plates were infected with VAC at a multiplicity of infection of 1 PFU/cell and incubated for 1 h at 37 °C and 5 % CO2. The cell monolayer was then washed with a selective serum-free medium and transfected with a recombinant integrative plasmid according to the following scheme: 3 μl of 1 μg/μl plasmid was mixed with 15 μl of 1 mg/ml Lipofectin; 1 ml of DMEM medium containing 25 μg/ml MPA, 250 μg/ml xanthine, and 15 μg/ml hypoxanthine was added and incubated for 15 min at room temperature; the resulting mixture was added dropwise onto the cell monolayer, and the cells were incubated at 37 °C and 5 % CO2. After 24 h, the medium was replaced with a selective maintenance medium (supplemented with 2 % FBS) and incubated under the same conditions for one day.

To enrich the viral progeny with the recombinant VAC variant, several passages (successive viral reproduction cycles in cells) were performed in 6-well plates with a selective medium. When a 90–100 % viral cytopathic effect (CPE) was achieved, the culture was frozen and thawed twice to destroy cells and release the virus in the culture medium. The resulting viral suspension was sonicated and further utilized for cloning using agarose overlay plaque assay under non-selective conditions (without addition of xanthine, hypoxanthine, and MPA to the DMEM medium). Several viral clones were selected and passaged under non-selective conditions in CV-1 cells until a 100 % CPE was achieved. The titers of resulting virus clone samples were determined in CV-1 cells as described previously (Yakubitskiy et al., 2015).

Viral DNA was isolated from individual clones using the QIAamp DNA Mini Kit (Qiagen, Germany) according to the manufacturer’s instructions. PCR analysis with the use of specific oligonucleotide primers (see Table 1) was performed to screen clones at viral genome regions containing either a target deletion or a nucleotide sequence insertion (Table 2).

**Table 1.**
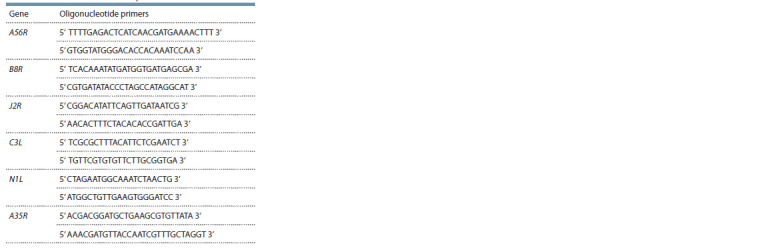
Oligonucleotide primers for PCR analysis of modif ied regions in the VAC genome used in the VACΔ6 vaccine development

**Table 2.**
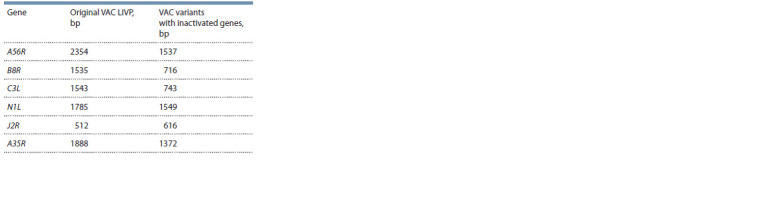
Lengths of PCR products for the original LIVP strain and VAC variants with inactivated genes

Clones with the disrupted target gene were selected based on PCR results (cloning round No. 1) and re-cloned using the agarose overlay plaque assay to prevent possible contamination with the original virus variant. Having conducted additional passages of the resulting subclones under non-selective conditions and achieved 100 % CPE of the cell monolayer, we performed a PCR analysis and selected clones with inactivated target gene (cloning round No. 2). Infectious titers of viral preparations were determined. The preparations were aliquoted, frozen, and used for further experiments.

VAC whole-genome sequencing. Sequencing was performed on a MiSeq instrument (Illumina, USA). For this, viral DNA was isolated from cell cultures infected with different viruses. The QIAamp DNA Mini Kit (Qiagen) was used for DNA isolation according to the manufacturer’s instructions.

Whole-genome sequence analysis. Nucleotide sequences of VAC variants were analyzed using software packages MIRA (v. 4.9.6), BWA (v. 0.7.15) (Li, Durbin, 2009), IGV (v. 2.3.78) (Robinson et al., 2011), Samtools (v. 1.3.1) (Li et al., 2009), Tabix (v. 0.2.5) (Li et al., 2009), and Genome- AnalysisTK (v. 3.6) (McKenna et al., 2010). Whole-genome sequences were aligned in Ugene Alignment Editor v. 1.24.1 (Okonechnikov et al., 2012) using the MAFFT algorithm (Katoh et al., 2002).

## Results

Introduction of targeted deletions/insertions into the VAC genome

**Fig. 1.**
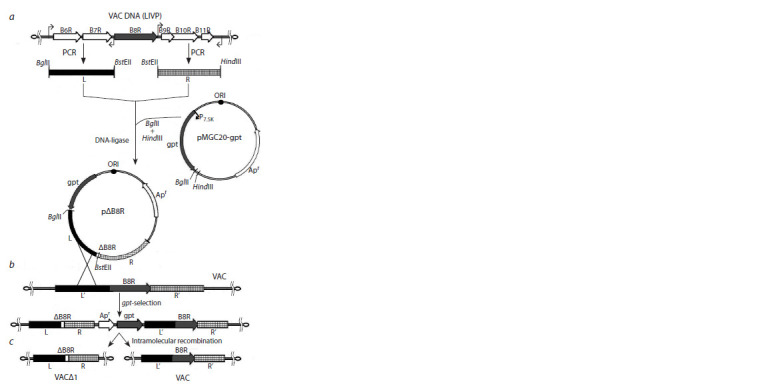
General scheme for obtaining VACΔ1 virus with a targeted B8R gene deletion а – scheme of hybrid pΔB8R plasmid design; b – recombinant insertion of the hybrid plasmid into the viral genome; c – division of the recombinant virus progeny into two variants after removal of selective pressure on the gpt gene (see explanations in the text).

We used transient dominant selection to obtain VAC with a targeted genome modification (Falkner, Moss, 1990). The integration/deletion plasmid (Fig. 1, a) carries a dominant selective marker (the gpt gene of Escherichia coli encoding xanthine-guanine-phosphoribosyl transferase, under the control of the 7.5K VAC promoter) located outside the extended regions of homology with the viral DNA flanking the disrupted/deleted gene (R stands for right, L stands for left).

**Fig. 2.**
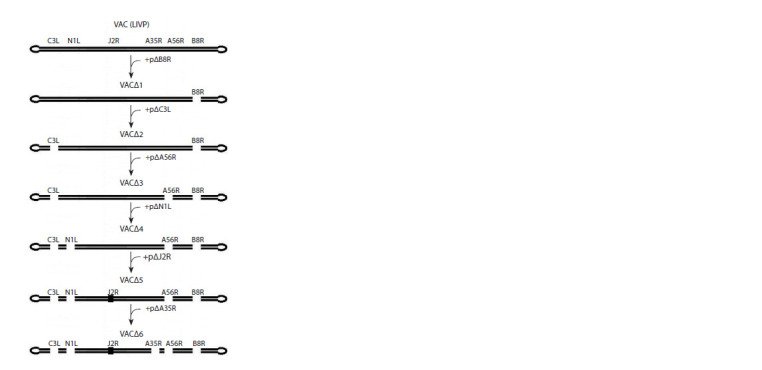
Scheme of recombinant VACΔ6 production

The bacterial enzyme xanthine-guanine-phosphoribosyl transferase, which is synthesized in mammalian cells, can restore purine nucleotide metabolism blocked by MPA. As a result of a single crossover between the integrative plasmid and viral DNA, a recombinant viral genome containing a fully integrated recombinant plasmid is formed (see Fig. 1, b). The genome contains both the gpt gene and long repeats R, R′ and L, L′. A genetic construct containing long repeats is unstable and can only exist under selective pressure on gpt. When selective conditions are removed (cultivation in a normal growth medium), intramolecular homologous recombination occurs in the viral genome (either R–R′ or L–L′); it yields two virus variants: viruses with either a disrupted or an intact gene (see Fig. 1, c). It should be noted that intramolecular recombination deletes all foreign sequences from the viral genome, which is important when creating VAC-based immunobiological preparations. In addition, cleavage of the entire plasmid sequence from the viral genome makes it possible to further produce double-, triple-, etc. recombinant viruses with disrupted loci in different genomic regions using the same technique and selective marker (Fig. 2).

The scheme for generating recombinant VAC variants with six inactivated virulence genes is presented in Fig. 2. The thymidine kinase gene in integrative plasmid pΔJ2R is disrupted by insertion of a synthetic DNA fragment; in other recombinant plasmids, target genes are deleted (Yakubitskiy et al., 2015, 2016).

A total of 18 serial passages were carried out in CV-1 cells when producing recombinant VACΔ6 from the LIVP strain: 6 serial passages under selective pressure (3–7 passages), 6 serial passages without selective pressure after the first cloning (3–4 passages), and 6 serial passages without selective pressure after the second cloning (2–4 passages) (Table 3).

**Table 3.**
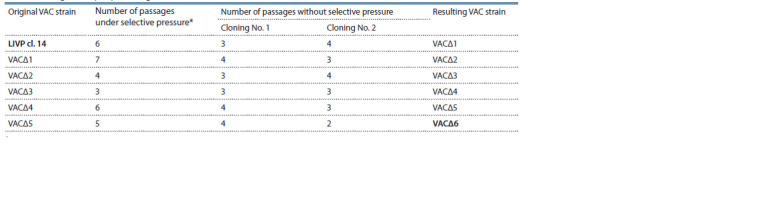
Passage history of producing the VACΔ6 strain from LIVP in CV-1 cells

Whole-genome sequencing of VAC variants

VACΔ6 passage history includes 71 passages in total, of which 31 and 40 passages were conducted under selective pressure on gpt and without selective pressure, respectively. Such a long passage history could cause additional off-target mutations in the VACΔ6 genome compared to the LIVP strain. Therefore, in order to assess possible off-target mutations occurring during transient dominant selection and the stability of both the original LIVP strain and its recombinant variants, we performed whole-genome sequencing of seven VAC strains (LIVP clone 14, VACΔ1, VACΔ2, VACΔ3, VACΔ4, VACΔ5, and VACΔ6).

As a result, seven whole-genome nucleotide sequences of the studied VACs were obtained; the genomic nucleotide sequence of the original clonal variant VAC LIVP was deposited in GenBank under the accession number KX781953.

A comparative analysis of complete viral genomes showed that, in addition to targeted mutations, only two nucleotide substitutions occurred spontaneously when generating VACΔ4 from VACΔ3; the mutations persisted in VACΔ5 and VACΔ6 genomes. Both nucleotide substitutions are located in intergenic regions (positions 1431 and 189738 relative to LIVP). Considering the total length of the viral genome (more than 190 kb) and a long passage history, this indicates the absence of off-target mutations in viral DNA when using transient dominant selection for obtaining recombinant VAC variants

A comparative analysis of complete viral genomes showed that, in addition to targeted mutations, only two nucleotide substitutions occurred spontaneously when generating VACΔ4 from VACΔ3; the mutations persisted in VACΔ5 and VACΔ6 genomes. Both nucleotide substitutions are located in intergenic regions (positions 1431 and 189738 relative to LIVP). Considering the total length of the viral genome (more than 190 kb) and a long passage history, this indicates the absence of off-target mutations in viral DNA when using transient dominant selection for obtaining recombinant VAC variants

The discovered stability of the VAC genome after 71 passages in CV-1 cells indicated the importance of using a more reasonable approach to confirm strain identity for the future largescale production of a VACΔ6-based vaccine. This method uses PCR with primers complementary to six regions of the viral genome at which the parental VAC LIVP strain was modified (see Table 1). Therefore, the genetic stability of the industrial strain VACΔ6 during multiple passages in 4647 cells (certified for cultivating the vaccine strain) was evaluated by PCR at preclinical stages.

A total of 15 serial cycles of 4647 cell monolayer infection with the industrial strain VACΔ6 and viral progeny production were carried out in accordance with the “Guidelines for Clinical Trials of Medicinal Products…” (2013). Viral DNA was isolated from both the original sample and 4647 cell cryolysate that had been infected with VACΔ6 obtained after the 14th passage. PCR analysis was performed using oligonucleotide primers shown in Table 1.

DNA isolated from intact 4647 cells and VAC LIVP DNA were used as a negative and positive PCR control, respectively

**Fig. 3.**
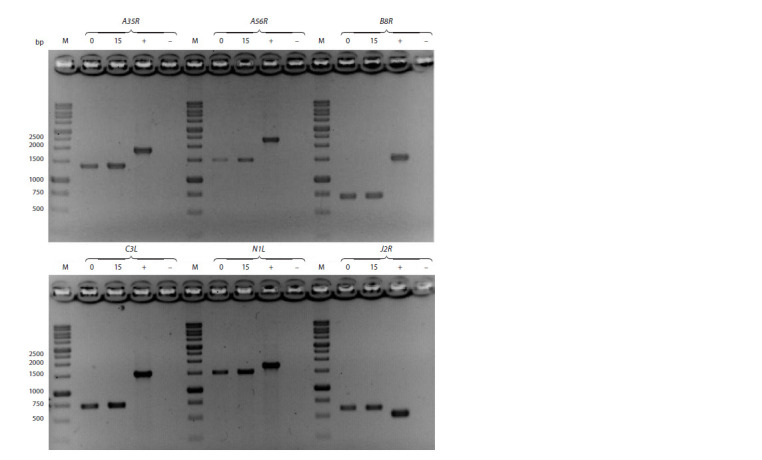
Electrophoretic separation of the DNA products of PCR analysis of the regions of viral genes A35R, A56R, B8R, C3L, N1L, and J2R 0 – PCR product obtained using genomic DNA of the original strain VACΔ6 as a template; 15 – PCR product obtained using genomic DNA of the VACΔ6 strain after 15 passages in 4647 cells as a template; M – DNA marker (length in bp is indicated on the left); “+” – PCR product obtained using VAC LIVP DNA; ”–” – negative control.

The theoretically calculated lengths of the DNA products of PCR performed with specific primer pairs are presented in Table 2. The electrophoretic separation of the resulting amplicons indicates that, after 15 passages of the industrial strain VACΔ6 in 4647 cells, the PCR products correspond to theoretically calculated values that do not differ from those of the PCR products obtained using DNA of the industrial strain VACΔ6 before passaging as the template (Fig. 3).

Sequencing of six DNA fragments corresponding to disrupted gene regions in VACΔ6 showed that all viral DNA sequences remained unchanged after 15 passages in 4647 cells.

## Discussion

The Vaccinia virus (VAC) is a member of the genus Orthopoxvirus of the family Poxviridae. Representatives of this genus are the largest mammalian viruses; their DNA genome contains about 200 genes. Poxviruses replicate in the cytoplasm of the host cells; the products of numerous viral genes control viral DNA replication, transcription, and translation of viral genes. In addition, many genes are involved in regulating the antiviral immune response, susceptible host range, pathogenicity, and other poxvirus properties. VAC is the most studied orthopoxvirus; it played a crucial role as a live vaccine in global eradication of smallpox (Shchelkunov, Shchelkunova, 2020).

Smallpox vaccines based on different VAC strains are moderately reactogenic. However, in mass vaccination, they can cause severe adverse reactions and even death in a small percentage of cases. Therefore, after confirmation of smallpox eradication in 1980, WHO strongly recommended to cease smallpox vaccination (Smallpox and its Eradication, 1988).

The cessation of vaccination resulted in the loss of the immune protection not only against smallpox but also other zoonotic human orthopoxvirus infections such as monkeypox and cowpox in almost all humans. Of particular concern is monkeypox, whose clinical manifestations in humans resemble smallpox. All of this has raised the issue of the need to develop new safe vaccines against orthopoxvirus infections using modern techniques.

We have implemented an approach to introduce targeted deletions into (inactivate) individual VAC virulence genes by genetic engineering without affecting viral replication in mammalian cells (Yakubitskiy et al., 2015). The protocol of transient dominant selection used for inactivation of each of the selected viral genes is carried out through serial passages and cloning of the virus in cells. The selection of generated VAC variants is based on PCR data in the target region of the viral genome. Before the advent of modern methods of wholegenome sequencing, the complete nucleotide sequence of the long VAC DNA was not controlled. Therefore, there was no answer to the question of how multiple VAC passages in cells can affect viral genome stability in general.

For the first time, we performed whole-genome sequencing of the original clonal variant VAC LIVP, the VACΔ6 vaccine strain, and five intermediate virus variants with a series of disrupted target genes using the obtained VACΔ6 strain with inactivation of six genes in different viral genomic regions as an example (see Fig. 2). A comparative analysis of complete viral genomes showed that, in addition to targeted mutations, only two nucleotide substitutions occurred spontaneously when producing VACΔ4 from VACΔ3; the mutations persisted in VACΔ5 and VACΔ6 genomes. Both nucleotide substitutions are located in intergenic regions (positions 1431 and 189738 relative to LIVP). These results show that transient dominant selection does not introduce significant off-target mutations into the viral genome. Apparently, this also indicates that the original VAC LIVP variant is suitable for reproduction in CV-1 cells and therefore stably maintains the genome integrity under experimental conditions

Cultivation of VAC, which is a mammalian virus, in heterologous primary avian cells (chicken embryo fibroblasts) seems to exert a high selective pressure on this virus and cause significant changes in the viral genome after multiple passages, which was observed in the MVA variant (Volz, Sutter, 2017).

In order to use VACΔ6 as a safe live vaccine for mass vaccination, one has to subject the resulting strain to multiple reproduction cycles in a cell culture certified for this purpose. Preservation of the attenuated phenotype/genotype of the vaccine strain is the most important criterion in large-scale virus production. For this reason, a total of 15 consecutive cycles of a 4647 cell monolayer infection with the industrial strain VACΔ6 were carried out in accordance with the “Guidelines for Clinical Trials of Medicinal Products…” (2012). The results of a PCR analysis and sequencing of six DNA fragments corresponding to the regions of disrupted VACΔ6 genes showed that viral DNA sequences in these regions remained unchanged after 15 passages in 4647 cells.

## Conclusion

Thus, the obtained results demonstrate high genetic stability of the studied recombinant strains with a long passage history in CV-1 and 4647 cells, which is an important positive characteristic of recombinant VACΔ6 variant as a stable vaccine strain for obtaining a fourth-generation live vaccine against smallpox and other orthopoxvirus infections.

## Conflict of interest

The authors declare no conflict of interest.
